# A New Iridoid Glycoside from the Roots of *Dipsacus asper*

**DOI:** 10.3390/molecules17021419

**Published:** 2012-02-03

**Authors:** De Ji, Chunfeng Zhang, Jingzhi Li, Haowei Yang, Jingyang Shen, Zhonglin Yang

**Affiliations:** State Key Laboratory of Natural Products and Functions, China Pharmaceutical University, Ministry of Education, 24 Tong Jia Xiang, Nanjing 210009, China; Email: njjide@163.com (D.J.); zhangchunfeng67@163.com (C.Z.); jingzhiyuyu@163.com (J.L.); yanghaowei2008@126.com (H.Y.); sjy898989@yahoo.com (J.S.)

**Keywords:** *Dipsacus asper*, iridoid glycoside, loganic acid ethyl ester, neuroprotective effect, Dipsaceae

## Abstract

A new iridoid glycoside, named loganic acid ethyl ester (**1**), together with five known compounds: chlorogenic acid (**2**), caffeic acid (**3**), loganin (**4**), cantleyoside (**5**) and syringaresinol-4′,4′′-*O*-bis-β-D-glucoside (**6**) were isolated from the roots of *Dipsacus asper*. The structure of compound **1** was elucidated on the basis of detailed spectroscopic analyses. Lignan is isolated from Dipsacaceae species for the first time. Compounds **1**, **4** and **5** had moderate neuroprotective effects against the Aβ_25–35_ induced cell death in PC12 cells.

## 1. Introduction

**Figure 1:**
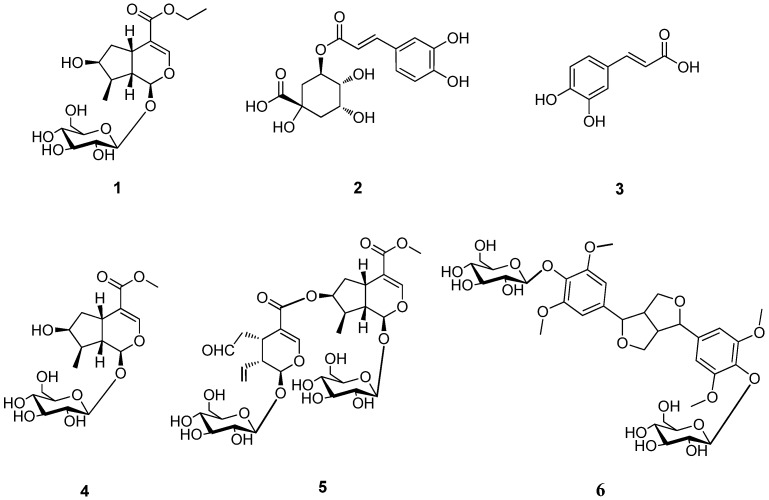
The chemical structures of compounds **1**–**6**.

*Dipsacus asper *Wall., (Dipsacaceae), is a perennial herb widely distributed in the southwest of the People's Republic of China. The roots of *D. asper* have been used in Traditional Chinese Medicine for hundreds of years as an antiosteoporosis, tonic and antiaging agent for the therapy of low back pain, traumatic hematoma, threatened abortion and bone fractures [1]. In previous studies, dozens of chemical constituents, including triterpene saponins, iridoids, phenolics and alkaloids have been identified from the roots of *D. asper* [2]. Pharmacological studies so far have demonstrated that, saponins isolated from the roots of this plant possess anticomplementary, antinociceptive, cytotoxic, osteoprotective, cardioprotective and inhibition of Alzheimer's disease activities, while phenolics possess neuroprotective and antioxidant effects [2]. Herein we report on the isolation from the roots of *D. asper* and structural elucidation of a new iridoid glycoside, together with five known compounds: chlorogenic acid (**2**) [3], caffeic acid (**3**) [4], loganin (**4**) [5], cantleyoside (**5**) [6] and syringaresinol-4′,4′′-*O*-bis-β-D-glucoside (**6**) [7] (Figure 1). Lignan is isolated from Dipsacaceae species for the first time. Compounds **1**, **4**–**6** were evaluated for their neuroprotective effects in PC12 cells against the Aβ_25–35_ induced cell death.

## 2. Results and Discussion

The air-dried roots of *D. asper* were pulverized and refluxed with 70% MeOH. The 70% MeOH extract was evaporated *in vacuo* to remove the MeOH and then diluted with H_2_O. The soluble fraction was subjected to column chromatography over macroporous resin HPD-722 and ODS to afford compounds **1**–**6**.

Loganic acid ethyl ester (**1**) was obtained as a white, amorphous powder. The TOF-MS gave a molecular ion at *m/z* 449.1818 ([M+HCOO]^−^), which corresponds to a molecular formula of C_18_H_28_O_10_. The IR spectrum (KBr) showed absorptions for hydroxyl (3420 cm^−1^), carboxyl (1688 cm^−1^), double bond (1634cm^−1^) and C–O–C (1076 cm^−1^) groups. The ^1^H-NMR spectrum of **1** showed one anomeric proton at δ_H_ 4.65 (d, *J* = 8.0 Hz), and together with the corresponding carbon resonances at δ_C_ 100.1, it was easily deduced that compound **1** contained a β-glucopyranose moiety. Meanwhile, the ^1^H-NMR spectrum of **1** showed the presence of an iridoid structure with one acetal proton at δ_H_ 5.27 (1H, d, *J* = 4.5 Hz), one characteristic H-3 proton at δ_H_ 7.38 (1H, s), one oxygenated methylene proton at δ_H_ 4.15 (2H, m), one oxygenated methine proton at δ_H_ 4.04 (1H, m), one methylene proton at δ_H_ 1.62 (1H, ddd, *J* = 5.0, 8.0, 14.0 Hz) and 2.24 (1H, ddd, *J* = 1.5, 8.0, 14.0 Hz,), three methine protons at δ_H_ 3.11 (1H, q, *J* = 8.0 Hz), 1.88 (1H, m) and 2.03 (1H, dt, *J* = 4.5, 9.0 Hz), and two methyl protons at δ_H_ 1.09 (3H, d, *J* = 7.0 Hz) and 1.27 (3H, t, 7.1). The ^13^C-NMR spectrum of **1** showed 18 resonances, including six for a sugar unit and twelve for an iridoid aglycone moiety.

**Figure 2:**
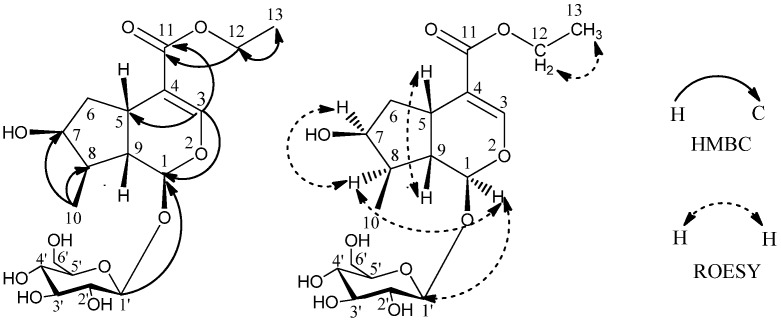
Key HMBC and ROESY correlations of **1**.

It is found that the NMR chemical shifts of **1** were very similar to those of the known compound loganic acid [8], which has 28 mass units less than **1**. Compound **1** might be an ethyl ester of loganic acid, as indicated by additional signals of δ_C_ 61.0 (C-12) and δ_C_ 14.6 (C-13), with the corresponding δ_H_ 4.15 (2H, m, H-12) and δ_H_ 1.27 (3H, t, 7.1Hz, H-13). HMBC correlations of H-13 (δ_H_ 1.27) with C-12 (δ_C_ 61.0) and H-12 (δ_H_ 4.15) with C-11 (δ_C_ 169.1) were also observed (Figure 2), which confirmed the above structure. The proton and carbon signals were assigned unambiguously using ^1^H, ^13^C, HSQC, HMBC NMR experiments.

The relative configuration of **1** was further determined by the NOESY experiment. The NOE correlations of δ_H_ 5.27 (H-1) with δ_H_ 1.88 (H-8), δ_H_ 3.11 (H-5) with δ_H_ 2.03 (H-9), δ_H_ 4.04 (H-7) with δ_H_ 1.88 (H-8) and no correlation between δ_H_ 1.88 (H-8) and δ_H_ 2.03 (H-9), revealed the relative configuration of **1** (Figure 2). Thus, the structure of **1** was elucidated as shown in Figure 1 determined as loganic acid ethyl ester.

Compounds **2**-**6** were identified as chlorogenic acid (**2**) [3], caffeic acid (**3**) [4], loganin (**4**) [5], cantleyoside (**5**) [6] and syringaresinol-4′,4′′-*O*-bis-β-D-glucoside (**6**) [7], respectively, by comparison of the spectroscopic data with those reported in the literature.

Zhang *et al*. found that *Dipsacus asper* extract significantly ameliorated animal’s performance impairment in the passive avoidance task and suppressed the overexpression of hippocampal Aβ immunoreactivity [9]. It has been demonstrated that the phenolics and saponins isolated from the roots of *D. asper* all possess neuroprotective effects [10,11], so the iridoids and lignan of compounds **1**, **4**–**6** were examined for their neuroprotective effects against the Aβ_25-35_ induced cytotoxicity in PC12 cells by the MTT method using salvianolic acid B as positive control [12,13]. The result showed that compounds **1**, **4** and **5** had moderate protective effects against the Aβ_25-35_ induced cell death (Table 1).

**Table 1:** Neuroprotective effects of the compounds **1**, **4**–**6** against the Aβ_25-35_ induced PC12 cell death.

Compound	Inhibition ratio (%) ^a^		
	10 μM	30 μM	100 μM
**1**	27.66 ± 1.72 ^b^	18.59 ± 1.10 ^b^	14.17 ± 0.38 ^b^
**4**	26.40 ± 2.05 ^b^	19.17 ± 1.34 ^b^	15.98 ± 2.11 ^b^
**5**	23.17 ± 1.87 ^b^	17.24 ± 0.87 ^b^	12.02 ± 1.46 ^b^
**6**	38.73 ± 2.01	35.24 ± 2.33	36.59 ± 2.33
salvianolic acid B ^c^	18.28 ± 1.02 ^b^	7.28 ± 0.87 ^b^	4.28 ± 0.58 ^b^

Results are presented as mean ± SEM (n = 3). ^a^ Inhibition ratio (%): 35.06 ± 0.86 (treated only with Aβ_25-35_, *p* < 0.01 compared with the control group); ^b^* p* < 0.01 compared with group treated only with Aβ_25-35_; ^c^ positive control.

## 3. Experimental

### 3.1. General

Optical rotations were measured with a JASCO P-1020 polarimeter. UV spectra were obtained on a Shimadzu UV-2450 UV-visible spectrophotometer. IR spectra were measured on a Bruker Tensor-27 spectrophotometer. NMR spectra were recorded at 303k on a Bruker AV-500 NMR (^1^H-NMR, 500 MHz; ^13^C-NMR, 125 MHz) instrument with TMS as internal standard, and chemical shifts were recorded as δ values. The 2D-NMR experiments, HSQC, HMBC and ROESY were performed using standard Bruker pulse sequences. The ESIMS experiment was performed on an Agilent SL G1946D single quadrupole mass spectrometer with an ESI source in negative-ion mode. the TOF-MS experiment was performed on Agilent orthogonal TOF/MS equipped with ESI source [drying gas, N2; flow rate, 9.0 L/min; temperature, 325 °C; nebulizer, 35 psig; capillary, 3,000 V; skimmer, 60 V; OCT RFV, 250 V; the sample was dissolved in MeOH; analyzed in negative-ion mode; fragment voltage, 120 V]. Column chromatography (CC) were performed on macroporous resin HPD722 (Cangzhou Bon Adsorber Technology Co., Ltd., China) and ODS (40–63 μm, YMC, Japan).

### 3.2. Plant Material

The air-dried roots of *D. asper* were collected from Lianshan County, Sichuan Province of China in July 2009, and authenticated by Professor Ping Li, Department of Traditional Chinese Medicine, China Pharmaceutical University. Voucher specimens (NO.20090701) are deposited in the State Key laboratory of Natural Products and Functions, China Pharmaceutical University.

### 3.3. Extraction and Isolation

The air-dried roots of *D. asper* (3 kg) were powdered and successively extracted two times with 70% MeOH (2 × 10 L) under reflux. After removal of the solvents *in vacuo*, the residue (456 g) was suspended in H_2_O (30 L) to afford H_2_O-soluble and H_2_O-insoluble fractions. The soluble fraction was chromatographied on a macroporous resin HPD722 column eluted with H_2_O, followed by step gradients with EtOH to obtain four fractions (Fractions 1–4). Fraction 1 (water eluent) was concentrated to subject to ODS column chromatography and eluted with MeOH–H_2_O (0:1–2:8) to afford compounds **2** (25 mg) and **3** (16 mg). Fraction 2 (20% EtOH eluent) was concentrated to subject to ODS column chromatography and eluted with MeOH–H_2_O (0:1–3:7) to afford compounds **5** (10 mg), **4** (24 mg), **6** (5 mg), and **1** (12 mg).

*Loganic acid ethyl ester* (**1**). White, amorphous powder; 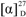 −86.3(c 0.082, MeOH); UV (MeOH) λ_max_ (log ε) 198 (4.07), 235 (4.36) nm; IR (KBr) γ_max_ 3420, 2933, 1688, 1634, 1399, 1288, 1076 cm^−1^; ^1^H-NMR (CD_3_OD) δ 5.27 (1H, d, *J* = 4.5 Hz, H-1), 7.38 (1H, s, H-3), 3.11 (1H, q, *J* = 8.0 Hz, H-5), 1.62 (1H, ddd, *J* = 5.0, 8.0, 14.0 Hz, H-6a), 2.24 (1H, ddd, *J* = 1.5, 8.0, 14.0 Hz, H-6b), 4.04 (1H, m, H-7), 1.88 (1H, m, H-8), 2.03 (1H, dt, *J* = 4.5, 9.0 Hz, H-9), 1.09 (3H, d, *J* = 7.0 Hz, H-10), 4.15 (2H, m, H-12), 1.27 (3H, t, *J* = 7.1 Hz, H-13), 4.65 (1H, d, *J* = 8.0 Hz, H-1′), 3.20 (m, H-2′), 3.30 (m, H-3′), 3.28 (m, H-4′), 3.37 (1H, m, H-5′), 3.66 (1H, m, H-6′a), 3.89 (1H, dd, *J* = 1.7, 12.0 Hz, H-6′b); ^13^C-NMR (CD_3_OD) δ 97.7 (C-1), 151.9 (C-3), 114.2 (C-4), 32.1 (C-5), 42.8 (C-6), 75.0 (C-7), 42.1 (C-8), 46.5 (C-9), 13.4 (C-10), 169.1 (C-11), 61.0 (C-12), 14.6 (C-13), 100.1 (C-1′), 74.7 (C-2′), 78.3 (C-3′), 71.6 (C-4′), 78.0 (1H, m, C-5′), 62.8 (C-6′); negative ESI-MS *m/z* 449 [M+HCOO]^−^; negative TOF-MS *m/z* 449.1818 [M+HCOO]^−^ (calcd for C_19_H_29_O_12_, 449.1817).

### 3.4. Neuroprotective Effects

Rat pheochromocytoma PC12 cell line, TCR 3, was obtained from the Committee on Type Culture collection of Chinese Academy of Sciences (CTCCAS, Shanghai, China) and grown in RPMI1640-based medium (Gibco, Gaithersburg, MD, USA) supplemented with 10% NBCS (Gibco). Cells were maintained at 37 °C in an atmosphere of 95% air/5% CO_2_ saturated with H_2_O. At 24 h after seeding in 96-well microplates, cells were incubated for 48 h with 15 μM Aβ_25-35_ (Sigma, St. Louis, MO, USA) in the absence or presence of the purified compounds. Cellular viability was evaluated by the MTT reduction assay. Briefly, 4 h after incubation with 0.5% MTT (20 μL) at 37 °C, the formazan crystals were lysed in 150 μL dimethyl sulfoxide (DMSO), and the microplates were shaken vigorously to ensure complete solubilization. The optical density at 570 nm (OD_570 nm_) was determined in a Microplate Reader (Dynatec Laboratories, Alexandria, VA, USA). All values were obtained from 6 wells and from 3 separate experiments. The inhibition rates (%) were calculated by comparing the treatment group with the tested compounds and the solvent control group. The percent of inhibition rate (%) = [1 − OD value of sample well/ OD value of control well] × 100.

## 4. Conclusions

In summary, three iridoid glycosides **1**, **4**–**5**, two phenolic acids **2**, **3** and a lignan glycoside **6** were isolated from the roots of *D. asper*. Lignan is isolated for the first time from Dipsacaceae species. Compound **1** was found to be a new iridoid glycoside by detailed spectroscopic analyses. Iridoid glycosides isolated from the roots of *D. asper* exhibited moderate neuroprotective effects against the Aβ_25-35_ induced cell death in PC12 cells. As the fraction containing compound **1** was obtained by using EtOH-H_2_O mixture, its presence could an artifact of the isolation process.
